# Shared probe design and existing microarray reanalysis using PICKY

**DOI:** 10.1186/1471-2105-11-196

**Published:** 2010-04-20

**Authors:** Hui-Hsien Chou

**Affiliations:** 1 Department of Genetics, Development and Cell Biology, and Department of Computer Science, Iowa State University, Ames, IA, 50011-3223, USA

## Abstract

**Background:**

Large genomes contain families of highly similar genes that cannot be individually identified by microarray probes. This limitation is due to thermodynamic restrictions and cannot be resolved by any computational method. Since gene annotations are updated more frequently than microarrays, another common issue facing microarray users is that existing microarrays must be routinely reanalyzed to determine probes that are still useful with respect to the updated annotations.

**Results:**

PICKY 2.0 can design shared probes for sets of genes that cannot be individually identified using unique probes. PICKY 2.0 uses novel algorithms to track sharable regions among genes and to strictly distinguish them from other highly similar but nontarget regions during thermodynamic comparisons. Therefore, PICKY does not sacrifice the quality of shared probes when choosing them. The latest PICKY 2.1 includes the new capability to reanalyze existing microarray probes against updated gene sets to determine probes that are still valid to use. In addition, more precise nonlinear salt effect estimates and other improvements are added, making PICKY 2.1 more versatile to microarray users.

**Conclusions:**

Shared probes allow expressed gene family members to be detected; this capability is generally more desirable than not knowing anything about these genes. Shared probes also enable the design of cross-genome microarrays, which facilitate multiple species identification in environmental samples. The new nonlinear salt effect calculation significantly increases the precision of probes at a lower buffer salt concentration, and the probe reanalysis function improves existing microarray result interpretations.

## Background

PICKY 1.0 [1] introduced several novel approaches to oligo microarray design for large and complex genomes. It went beyond sequence comparison and utilized efficient thermodynamic calculations in a whole genome scale to determine the quality of all probe candidates. It also employed a global optimization strategy to ensure the entire microarray, not just individual probes, are optimized for best sensitivity, specificity and uniformity. Since PICKY can run on all major computing platforms and is computationally efficient, it has been used by several research groups to design their oligo microarrays [2-10]. In particular, the rice microarray project sponsored by National Science Foundation selected PICKY to design its whole genome rice microarrays and the *Xanthomonas oryzae *dual rice pathogen microarray [10-12]. Independent evaluations of microarray design software indicated that PICKY generates quality probes [13,14]. The results from a recent quantitative evaluation showed that PICKY-designed microarray probes are robust and consistent throughout a wide range of temperature and sample concentration [15]. In this article we describe some new features added to PICKY since version 1.0 release and their algorithmic details; these new features make the latest PICKY 2.1 more versatile to microarray users.

A primary difficulty of microarray design for large genomes, like those of rice or maize, is their large gene families. Each gene family contains many highly similar genes that are thermodynamically indistinguishable to microarray probes. For example, the largest transposon gene family in rice contains over 9399 sequences that are more than 90% similar to each other over 90% of their entire length [16]. Probes designed to detect genes in this family likely will bind to multiple targets -- the keys are how to rationally determine the intended targets and to also avoid all unintended targets. A subset of genes that share common sequence regions may be rolled into a group to share a common probe; these common regions, however, are often highly similar to regions contained in the other genes. The conflicting needs are to target some common regions for shared probe design and to also avoid the other common regions to prevent cross-hybridizations. Therefore, it is more difficult than single-target probe design to choose a probe that can be shared but does not cross-hybridize with the other nontarget genes existing in the same gene family. The "shared probe" design feature has been added to PICKY; it allows genes in a group to be studied as a whole if not individually. This new feature differs from previous methods to discover "non-unique probes" [17,18]: the shared probes are not ranked by sequence level comparisons (e.g., their longest common substring with nontargets) but by their thermodynamic comparisons with targets *and *nontargets, any number of genes can share the same probe, and a gene can share different probes with different sets of genes. Generally, an algorithmic method to design probes shared by a few genes is more desirable than the absence of any probe to detect any of the genes. Note that some genes sharing a probe may also acquire their own unique probes with different target regions; thus, the identify of genes detected by a shared probe is often resolvable when considering multiple probes. The division of gene families into groups by PICKY is entirely computational and may not necessarily reflect their evolutionary distances.

A recently published biochemistry study demonstrated that the salt effect on DNA annealing stability is generally nonlinear, in contrast to the linear salt effect correction commonly used in melting temperature estimation equations [19]. Nonlinear salt effect suggests that the optimal microarray probes at different hybridization salt concentrations may not be the same. A more precise nonlinear salt effect calculation is added to PICKY to enhance the precision of the designed probes under a specific microarray protocol with a known salt concentration. Unlike the linear salt effect calculation which depends only on salt concentration, the nonlinear salt effect calculation also depends on DNA context. Therefore, new code is added to the PICKY design algorithm to keep track of the DNA binding context for each probe candidate.

Another frequently occurring issue is that microarrays can seldom keep up with the rapid progress of sequence annotation updates. For example, the NSF 45K rice microarray was designed using gene models from version 3 of the Rice Genome Annotation [16]. This microarray is still being used by many users, but three newer rice annotations have been released [20]; the latest version 6 annotation has much improved gene models [21]. It is impractical to keep making new microarrays each time the sequence annotation gets updated. Although most probes on the existing rice microarrays should continue to work, some of the probes may no longer function as expected due to conflicts with the newer gene models. It is possible to add new probes to an oligo microarray for newly discovered gene sequences, but it incurs extra cost that should be minimized. A new feature is added to PICKY to reanalyze existing probes against new sequence information to determine probes that are still valid. Invalid probes can be ignored during subsequent data analysis although they cannot be removed from printed microarrays. Only genes, new or old, that no longer have valid probes to detect them will need new probes to be added during the next microarray print. Therefore, the reanalysis feature reduces the cost of microarray update and maintains microarray quality. This feature may also be used to examine vendor supplied microarrays against users' gene sets to include only valid probes for data analysis, even if different gene sets have been used to design the microarrays.

## Implementation

### Shared probe design

To design shared probes, there are three requirements: 1) to be able to efficiently determine common regions among input sequences that are long enough to be targeted by probes; 2) to be able to efficiently distinguish these common regions from other highly similar regions during probe design so they would not be considered as nontargets and prevent probe targeting; and 3) to be able to thoroughly examine the thermodynamic characteristics of probe candidates targeting these common regions to prevent them from cross-hybridizing with nontargets in the whole genome. Although it is relatively easy to achieve the first requirement, it is harder to achieve the second and third requirements because common regions among gene family members often vary in their similarity levels -- a slight difference in their mutual similarity can mean either good targets for shared probes or very detrimental nontargets for shared probes.

**Figure 1:**
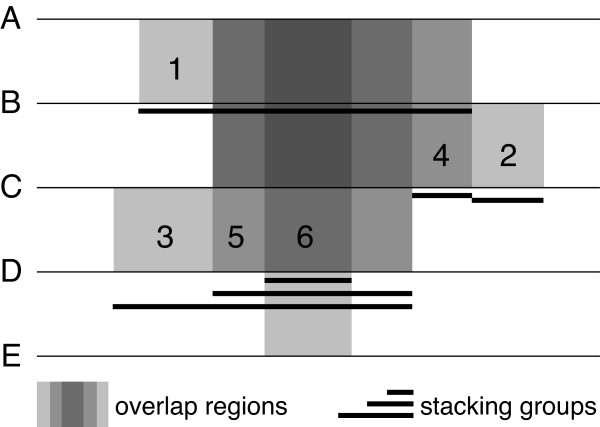
**An example of overlapping gene family sequences**. Five sequences A--E can overlap each other in six regions as indicated by the gray colors; darker grays indicate more sequences that overlap. These common regions are represented by suffix groups, which are found on the suffix array and hosted by sequences with the black underlines (i.e., sequences B, C and D). The underlines also indicate the stacking of the suffix groups when a host sequence is being processed.

Although it is straightforward to detect common regions between any two sequences, from the perspective of probe design these common regions often randomly overlap, making it difficult to target a probe. An example helps illustrate the complexity. In Figure 1, five sequences A-E are shown to share six common regions. Region 1 is shared by A and B, region 2 is shared by B and C, region 3 is shared by C and D, region 4 is shared by A-C, region 5 is shared by A-D, and region 6 is shared by all sequences. The common regions overlap each other, e.g., region 4 overlaps regions 1 and 2, and region 6 overlaps all other regions. Therefore, a common region may be implicitly divided into more regions, and probes targeting the region should not cross any of its dividing boundaries set by the other regions. For example, probes designed to target the early part of region 1 are shared only by sequences A and B, but probes designed to target region 5, which overlaps region 1, are shared by sequences A-D. A probe that targets the boundary area between regions 1 and 5 does not make a good probe for either region. This example demonstrates that simply finding a long common region is not sufficient to design good shared probes.

Many indexing data structures can be used to efficiently discover all common regions among input sequences [22]. Since suffix array is the chosen data structure in PICKY, we focus on some of its well-known properties. One property is that suffixes sharing the longest common prefixes must be collated into the same *group *on the suffix array. In addition, different such groups must be separated by groups of shorter common prefixes that may contain as few as just one suffix. Since the longest common prefix (LCP) array has already been constructed as part of PICKY computation [23], the following algorithm suffices to achieve the first requirement, to find all common regions long enough to accommodate probes:

(1) Scan the LCP array, and locate groups of suffixes whose LCP values are greater than the minimum probe size.

(2) For each of the groups found, check if any of the following conditions is true:

1. Suffix(es) from a nontarget input sequence or the reverse complement of any input sequence is in the group.

2. Either one of the LCP values bordering the group is greater than the maximum allowable length of exact nontarget match.

(3) If either condition above is true, this group is invalid and is skipped. Otherwise, record it in a lookup table indexed by the left-most sequence in the group.

**Figure 2:**
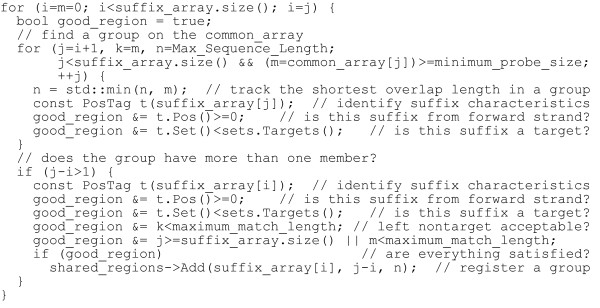
**Example implementation to discover all common region groups that can accommodate probes**. suffix_array and common_array are always the same size; i and j are the left and right boundaries of an identified common region group; k saves its left overlap length with nontargets; m saves its right overlap length with nontargets; and n holds the shortest common region within the group.

Step 1 discovers all regions that may accommodate probes. PICKY allows users to provide a list of nontarget sequences to be avoided during the microarray design; these can be any transcripts that might be encountered by the microarray (e.g., mitochondrial RNA). PICKY also considers the reverse-complements of all input sequences to be nontargets; this prevents secondary structure formations on the probes or on their targets. The details of these are described in the PICKY 1.0 paper [1]. If suffixes from nontarget sequences or the reverse-complements of any sequences are in a group, the group cannot be used to design probes. If a group is bound on either side by overlaps longer than the maximum allowable length of exact nontarget match, then suffixes in the group are overlapping too much with nontarget sequences, thus the group cannot be used either. The probe size and the maximum length of nontarget match are user specified parameters. In the algorithm, steps 1 and 2 can be combined in implementation and run in linear time. Step 3 can run in either constant time or logarithmic time depending on whether a hash table or a balanced binary tree is used for the lookup table. The worst-case complexity of this algorithm is thus *O(n *log *n)*, where *n *is the number of suffixes from all input sequences (i.e., the total bases). Figure 2 presents an example implementation of this algorithm in C++.

The left-most sequence that is used to record a group is called the *host sequence*. The *span *of a group is defined to be its number of members, and the *length *of a group is the shortest overlap (i.e., the smallest LCP value) among all its members. In Figure 1, the common regions are represented by the groups hosted on sequences B, C and D as indicated by their thick underlines. In particular, sequence D hosts three different groups representing regions 3, 5 and 6 with span value 2, 4 and 5, respectively. It is necessary to track the stacking of these groups so probes can be shared by the correct member of sequences and no probe may cross different groups. In Figure 1, probes targeting the first part of region 3 can only be shared by sequences C and D, but probes targeting region 5 or 6 can be shared by more sequences. No probe, however, should target any of the stacking group boundaries. The following algorithm efficiently traverses all host sequences and tracks their stacking groups:

(1) Iterate through all host sequences on the lookup table.

(2) Sort groups on a host sequence based on their start position, and then push the very first group onto a stack.

(3) While the stack is not empty, do one of the following:

1. If there are no remaining groups for the host sequence, pop the group on top of the stack, process it and add its probe candidates to its probe priority queue.

2. If the start position of the next group overlaps the right end of the group currently on top of the stack with at least maximum nontarget match length, process the region of the stack-top group up to the beginning of the next group, add its probe candidates to its probe priority queue, and then push the next group onto the stack.

3. If neither of the above is true, pop the group on top of the stack, process it, and add its probe candidates to its probe priority queue. If the stack is now empty, push the next group onto the stack.

To "process" a group region, we meant that the corresponding region of the group on the host sequence is used to identify probe candidates; this will be discussed shortly. The time to sort groups in step 2 is negligible, because only distinct groups long enough to accommodate probes are recorded and few are expected per each host sequence. The three choices in step 3 always advance some distance on a host sequence or reduce the stack size. Therefore, without counting the time to process a group region, this tracking algorithm takes linear time to run in practice.

**Figure 3:**
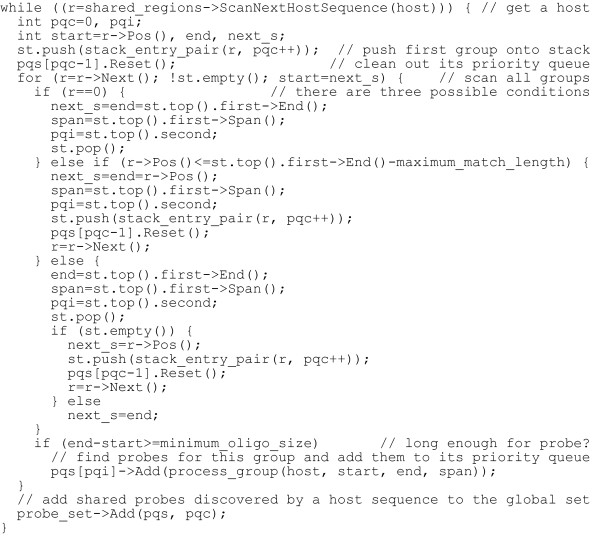
**Example implementation to traverse all host sequences, track their stacking groups and process the groups for shared probe design**. r points to each overlap group on a host sequence, which contains four data fields used in this algorithm: Pos, the start of the group on the host sequence, End, the end of the group on the host sequence, Span, the span value of the group, and Next, pointer to the next group; host is the host sequence currently being scanned; pqc counts the total number of distinctive groups on a host sequence; pqs is a collection of priority queues for each associated group; start and end indicated the range of the current group being processed; span records its span value; and next_s is the start position of the next group. Each stack entry in st contains a pair of values: the first is the r pointer to a region as described above, and the second is the pqi index into pqs for storing shared probes designed for a group.

In step 3.2, if the next group overlaps at least maximum nontarget match length with the group currently on top of the stack, then the right end of the next group cannot extend beyond the right end of the stack-top group. In this case we say that the next group *covers *the stack-top group. To prove this is always true, assume the opposite that a next group goes beyond the right end of the stack-top group but overlaps it with maximum nontarget match length. Suffixes of the stack-top group cannot all be also members of the next group because that would extend the stack-top group to the right end of the next group and contradict our assumption. Therefore, suffixes of the stack-top group that are not members of the next group must have overlapped at least the maximum nontarget match length with suffixes of the next group, which prevents the next group from even being added to the lookup table based on step 2.2 of the first algorithm. Therefore, a group that covers another group must be entirely within the region of the covered group and must have more members (i.e., a larger span value) than the covered group. A priority queue and stack combination can be used to keep track of the various groups on a host sequence: a priority queue is associated with each group and is used to store and prioritize its best probe candidates, while a stack is used to keep track of all stacking groups. For example, host sequence D in Figure 1 contains three stacking groups. When the group representing region 6 is being processed, the groups for regions 3 and 5 are pushed down the stack as indicated by the underlines. Processing on the host sequence never backtracks; thus, after group 6 has been processed, only the remaining region of group 5 will be processed. Group 3 will later be skipped because the remaining region of group 3 becomes zero after group 5 has also been processed. Figure 3 presents an example implementation of this algorithm in C++.

The final requirement is to process each group region, identify all shared probe candidates and examine their thermodynamic characteristics, to make sure that only probes with the least possibility to cross-hybridize with nontargets will be chosen. Most of this requirement has been solved in PICKY 1.0 for unique probe design [1]. PICKY 1.0, however, considers all common regions detrimental. Longer ones, as those encountered during shared probe design, will immediately rule out probe candidates targeting those regions. The PICKY algorithm has been modified to accommodate the shared probe design feature. Now, when processing each group region, the same algorithm for unique probe design is used but the known span value of each group prevents group members from being identified as nontargets. Therefore, only true nontargets will be screened during the shared probe design process. This modification finally achieves basic requirement 3.

Although a large gene family is expected to contain many common regions and produce many shared probe candidates, most of the candidates are not useful because they often imperfectly match some highly similar nontargets in the same gene family. For example, among the 61420 gene models used to design the NSF 45K rice microarray [12], 54761 common regions were identified, which produced 26519 distinct shared probe candidates. After thermodynamic screening, however, only 3214 shared probes can be chosen. This example reveals the importance of thermodynamic screening especially for shared probe design -- a sequence comparison method can identify the 26519 probe candidates but does know many of them may cross-hybridize with nontargets.

As seen in Figure 1, a common region may be represented by several disjoint groups and stacking groups (e.g., region 2). Groups that stack on each other may not necessarily be recorded by the same host sequence and may not all be visited during the processing of any particular host sequence (e.g., groups for regions 1, 4 and 5 stack but are on three different host sequences). Nevertheless, due to the insufficient span value of a covered group, the covered region of the group always triggers a maximum nontarget match length violation and is efficiently skipped during probe design. The covering groups with higher span values will then cover (or may have already covered) the skipped region when their host sequences are being processed. In Figure 1, when host sequence B is being processed, the group 1 region covered by groups 5, 6 and 4 is skipped because the span value of group 1 is 2 and it cannot prevent extra members in the other groups from being identified as nontargets. Thus, only in the early part of group 1, which is not covered by any other groups, can shared probes be designed only for sequences A and B using this host sequence.

### Nonlinear salt effect calculation

PICKY uses the nearest neighbor (N-N) parameters determined by biochemists in standard buffers to estimate the melting temperature between two DNA molecules [24-27]. Standard buffers have a salt [Na^+^] concentration of 1 M. Most microarray hybridization buffers, however, contain less salt. To accommodate the difference, a salt effect correction is added to the equation that calculates the melting temperature. At the time of PICKY 1.0 publication, the prevailing appearance of the correction took a linear form in the middle of the following equation:(1)

After PICKY 1.0 publication, a comprehensive study demonstrates that the salt effect on DNA melting temperature is generally nonlinear; thus, the commonly used linear salt effect correction predicts melting temperatures that can significantly deviate from the measured values [19]. A higher melting temperature prediction error can throw off microarray design precision and reduce the quality of microarray data. This new study suggested that salt effect should be described using a nonlinear equation:(2)

This new equation uses two terms to correct for salt effects: a linear term which depends on both salt concentration and sequence binding context, and a quadratic term which depends only on salt concentration. The *gc *in the linear term is the GC content of two binding DNA molecules, and it makes the salt effect calculation context-sensitive. All other terms in the two equations above are explained in the cited literature. PICKY 2.1 incorporates this new equation and offers both equations for microarray design. If the nonlinear equation is chosen, PICKY has to dynamically maintain the GC content in its innermost loop of calculation, thus it runs about 1/3 slower than the time it takes when the linear equation is chosen.

**Figure 4:**
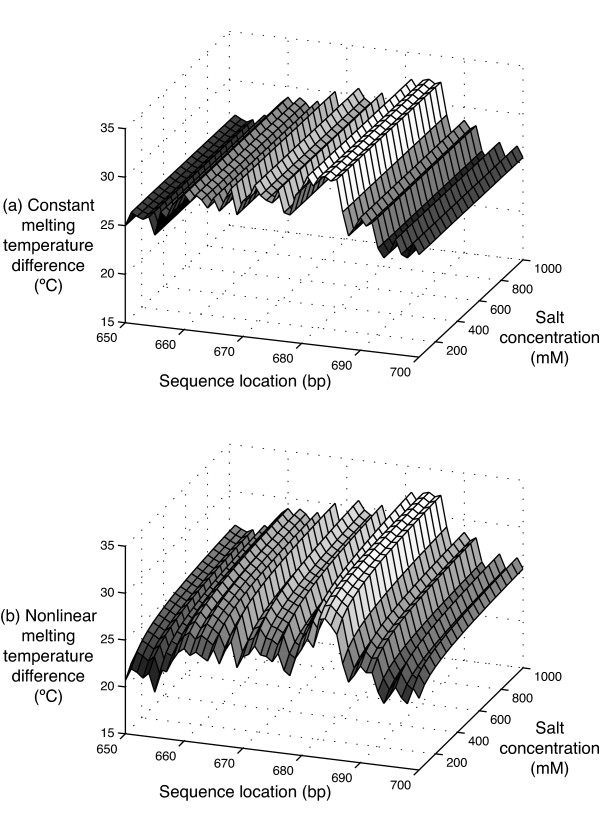
**A comparison of linear and nonlinear salt effects**. The target and closest nontarget melting temperature differences of 50-mer probes calculated using (a) the linear salt effect equation and (b) the nonlinear salt effect equation are expressed as a function of targeting sequence locations and salt concentrations. For example, the 50-mer probe targeting location 650-699 under 0 salt concentrations has a calculated melting temperature difference of either 25°C or 21°C using the two different equations. The temperature difference exhibits no dependence on salt concentration when calculated using the linear salt effect equation but becomes sensitive to salt concentration when calculated using the nonlinear salt effect equation.

Using the linear equation, PICKY always produces the same set of probes at different salt concentrations, because the *difference *between the linearly predicted target and nontarget melting temperatures is the same even though their *values *are changing with salt concentrations. PICKY optimizes its design by selecting probes that can achieve the highest melting temperature differences. Because the salt effect term in the linear equation depends only on salt concentration, it is canceled out when two melting temperatures predicted at the same salt concentration are compared. For example, when PICKY is used to design probes for a set of 3460 maize sequences, the same set of 3352 probes is generated at different salt concentrations. It is shown under the "Linear Probes" column in Table 1. This independence on salt concentration can also be seen in Figure 4a, where the melting temperature differences between targets and nontargets of 50-mer probe candidates targeting different locations on a sequence are calculated using the linear equation: the temperature differences change with target locations, but not with salt concentrations.

**Table 1:** Comparison of design results using the linear and nonlinear salt effect equations at different salt concentrations.

**Salt Conc**.	Linear Probes	Nonlinear Probes	Same Probes	Overlap Probes	Average Temperature Difference	Medium Temperature Difference
50	3352	3254	1020	2380	2.70	2.38
100	3352	3281	1301	2501	1.15	0.62
200	3352	3310	1662	2630	1.80	1.37
300	3352	3321	1977	2809	1.80	1.55
400	3352	3326	1734	2307	2.53	1.67
500	3352	3332	2253	2780	1.75	1.35
600	3352	3339	2333	2754	1.62	1.12
700	3352	3340	2409	2698	1.46	0.86
800	3352	3342	2475	2668	1.25	0.58
900	3352	3342	2544	2633	1.01	0.29
950	3352	3344	2565	2617	0.89	0.14
1000	3352	3352	3352	3352	0.00	0.00

Salt concentrations are shown in milliMolar (mM). The "Linear Probes" are the same but the "Nonlinear Probes" vary greatly at each salt concentration. Comparing the two probe sets, the "Same Probes" and "Overlap Probes" columns show the number of their probes that are the same or overlapping. Although at each salt concentration the "Average" and "Medium" target melting temperatures of the two probe sets differ just slightly, the two probes sets do not converge until reaching the 1000 mM standard salt concentration level.

The nonlinear salt effect equation induces the dependence of PICKY probe design on salt concentration. As seen in Figure 4b, the melting temperature differences predicted by this equation for the same 50-mer probe candidates now depend not just on target locations but also on salt concentrations. As a result, the probe sets selected by PICKY at different salt concentrations vary greatly; they are summarized under the "Nonlinear Probes" column in Table 1. At each salt concentration, the "Same Probes" and "Overlap Probes" columns in Table 1 compare the probe sets obtained using the linear and nonlinear equations; they show the number of probes between the two sets that are the same or overlap somewhat. About a quarter probes are still different between the two probe sets even at the salt concentration of 950 mM which is very close to 1 M. It can be seen in Table 1 that the average and medium predicted target melting temperatures of the two probe sets differ within just 1~2°C throughout the salt concentration range, but probes in the two sets only converge at the 1 M standard buffer salt concentration. This is because the nonlinear salt effect equation locally influences individual probe selections under different salt concentrations, although it predicts roughly the same melting temperature average as the linear equation predicts.

### Reanalysis of existing probes

PICKY 2.1 incorporates another new feature to map existing probes against any gene sets and evaluate their thermodynamic properties. There are many applications of this new feature, e.g., to evaluate third party microarray design quality, to characterize existing microarrays against newly annotated gene sets, and to determine PCR primer specificity. To efficiently map probes, PICKY computes a prefix index table during the construction of the suffix and LCP arrays of the input sequences. This table divides the suffix array into smaller regions that can be independently searched using a binary search algorithm on suffix array [22]. PICKY tries to first locate a suffix that contains a query probe as a prefix, and then expands from the match site to discover all such suffixes. As stated earlier in explaining the shared probe design algorithm, all suffixes sharing the same probe prefix must be collated into the same group on the suffix array, so the expansion is local and efficient. The complexity of the probe mapping algorithm is expected to be *O*(*m*+log *n*/4^x^), where *m *is the probe length, *n *is the total sequence bases, and *x *is the index prefix length.

Several different outcomes may result for a probe being mapped: it may not be found to target anything in the input sequences; it may be found to target only the user supplied nontarget sequences; or it may be found to target some sequences but also perfectly match some user supplied nontarget sequences or the reverse-complements of any sequences. In all of these cases, the probe is not considered useful. Once a probe is mapped onto target sequences and is not found to exactly match nontargets, its thermodynamic characteristics are evaluated by PICKY. If its target and nontarget melting temperature difference is less than a minimum value set by the users (e.g., 15°C), then the probe is not specific but may still be usable. Finally, a probe may be found to target multiple sequences, i.e., it can be a shared probe. PICKY will sort the query probes based on their classifications and present them on screen using different color coding to indicate their types. A complication is that some probes may overlap each other on their target sites so their colors might be mixed on the screen display; the textual output generated by PICKY unambiguously describes the type of each query probe disregard whether it overlaps with the other probes or not.

## Results

**Figure 5:**
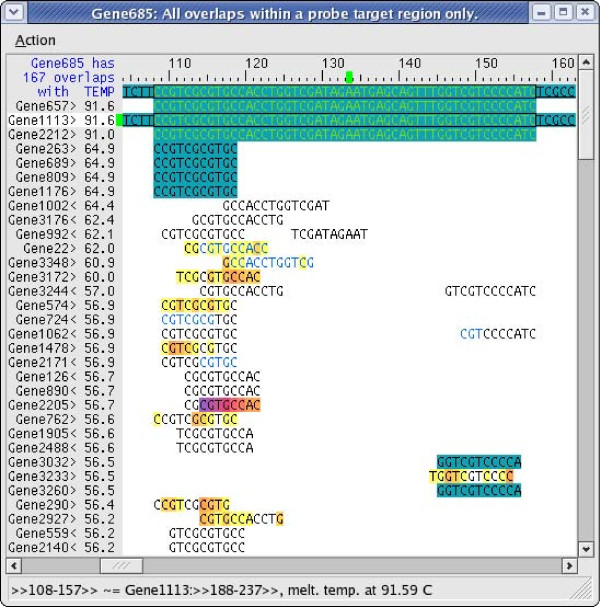
**An example of sequences sharing the same probe**. In this probe target region view on Gene 685, Genes 657, 1113 and 2212 are collected at the top and shown to contain the same target region. The shorter DNA fragments below them are detected nontargets to the probe. When a fragment is moused over, PICKY dynamically displays an alignment of the fragment-containing sequence (e.g., Gene 1113 as shown) with the target sequence (i.e., Gene 685). The melting temperatures of the probe with all its targets and nontargets are shown in the TEMP column and are used to sort the list.

The shared probe design requires a new graphical user interface and output file format to convey the information. Figure 5 shows an example of how such information is displayed by PICKY. The target region of a shared probe on Gene 685 is being viewed. Three additional Genes 657, 1113 and 2212 are also being targeted by the same probe. This can be seen from their matching green DNA characters. Because of dangling end differences [28], their estimated melting temperatures with the shared probe may be slightly different (e.g., Gene 2212). All detected nontargets of the shared probe are also listed after the four targets and are sorted based on their estimated melting temperatures with the probe. The closest nontarget Gene 263 has an estimated melting temperature of 64.9°C which is significantly lower than the 91°C of the targets. The PICKY design strategy is to widen the melting temperature differences between targets and nontargets, and this is also true for shared probes. When users move their mouse pointer over each of the matching fragments, an expanded alignment beyond the probe target region is dynamically produced to show the similarity between the gene being targeted (i.e., Gene 685) and the other gene (e.g., Gene 1113) at the matching site. There are several other views of PICKY computation results that are documented in the PICKY user guide. The probe reanalysis results are displayed using the same interface, but if a mapped probe is not specific, its nontarget list can be very long and the estimated nontarget melting temperatures can be very high. Although all nontargets are detected and analyzed, to conserve disk space, the PICKY output file reports only the closest nontarget of each probe, i.e., the one with the highest estimated melting temperature with the probe. Each target sequence sharing the same probe is then listed in the output. The probe reanalysis output also uses the same file format, but some queried probes may not be found to target any sequence.

As reported earlier, PICKY has been used by many research groups to design their microarrays [3-10]. As part of its quality assurance testing, we have also used it to design microarrays for 13 model species. Selected results are compared in Table 2. Similar to many optimization problems, tradeoffs can be made during microarray design. One tradeoff is between the specificity of the probes and the number of probes designed. Given different design constraints, PICKY can design probe sets that are significantly different in size. For example, using its stringent default parameters, PICKY can only find 9134 probes for the 28205 genes in a human gene set; with somewhat relaxed design parameters, PICKY can then find 25080 probes for almost all genes in the human gene set. PICKY has more than 15 design parameters, but the ones that most influence probe specificity are the minimum melting temperature difference between targets and nontargets and the maximum allowable exact nontarget match length. If the minimum temperature difference is lowered or the maximum nontarget match length is increased, PICKY is allowed to choose from more probe candidates that would have been automatically ruled out by more stringent settings. The detail discussion of PICKY parameters and how they influence its design output is beyond the scope of this article and is provided in the PICKY built-in help system. The gene sets used to design probes for the 13 model species and the PICKY parameters used to design each probe set reported in Table 2 can be found on the PICKY website [29].

**Table 2:** PICKY execution time and result under two different design constraints.

Model species	Gene set size	More sensitive design	More relaxed design
***E. coli***	5317 genes 4 848 788 bp	2-CPU: 0 h 9 m 4651 probes	4-CPU: 0 h 7 m 4852 probes
**Bee**	11 324 genes 6 022 273 bp	2-CPU: 0 h 11 m 10 237 probes	4-CPU: 0 h 17 m 10 995 probes
**Yeast**	6702 genes 9 081 699 bp	2-CPU: 0 h 22 m 5892 probes	2-CPU: 0 h 35 m 6164 probes
***P. falciparum***	9518 genes 10 749 024 bp	2-CPU: 0 h 4 m 4199 probes	4-CPU: 0 h 55 m 7481 probes
**Zebrafish**	12 238 genes 23 015 888 bp	4-CPU: 0 h 22 m 9535 probes	2-CPU: 3 h 2 m 10 749 probes
***Drosophila***	18 962 genes 32 217 720 bp	2-CPU: 1 h 2 m 12 611 probes	4-CPU: 2 h19 m 15 686 probes
**Chicken**	26 236 genes 32 759 147 bp	4-CPU: 0 h 23 m 15 931 probes	2-CPU: 4 h 21 m 22 509 probes
***C. elegans***	30 935 genes 34 783 951 bp	2-CPU: 0 h 41 m 16 887 probes	4-CPU: 3 h 37 m 25 602 probes
***Arabidopsis***	28 952 genes 36 327 482 bp	2-CPU: 1 h 3 m 18 297 probes	2-CPU: 8 h 26 m 26 608 probes
**Maize**	58 579 genes 39 022 169 bp	2-CPU: 0 h 48 m 21 993 probes	2-CPU: 8 h 9 m 48 620 probes
**Mouse**	35 284 genes 68 639 601 bp	4-CPU: 0 h 23 m 11 450 probes	4-CPU: 9 h 36 m 28 435 probes
**Human**	28 205 genes 72 748 721 bp	4-CPU: 0 h 19 m 9134 probes	4-CPU: 8 h 58 m 25 080 probes
**Rice**	61 251 genes 94 194 626 bp	2-CPU: 10 h 1 m 39 094 probes	4-CPU: 10 h 33 m 43 376 probes

**Figure 6:**
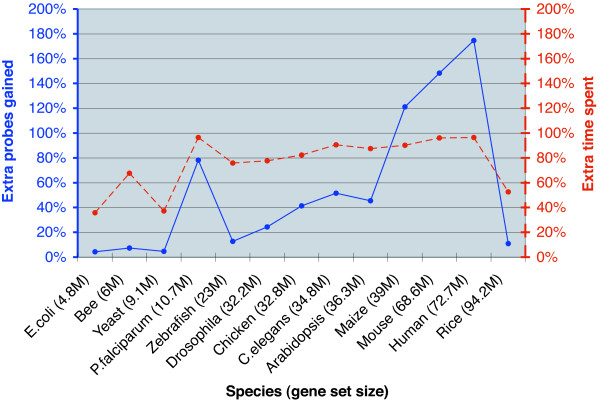
**An information-theoretical comparison of genome complexity**. Relative increases of PICKY computation time and additional probes it can find for the 13 model species are shown. When its design constraints are relaxed, PICKY has to compare more probe candidates against nontargets to decide whether these probe candidates can correctly identify their targets. A genome is considered more complex if the extensive thermodynamic comparison identifies more distinguishable gene sequences that can be targeted by microarray probes. For maize, mouse and human, more probes can be gained than the extra time spent to calculate them, which suggests that these species may have more complex genomes.

Two probe sets are designed for each of the 13 model species. Under "More sensitive design", fewer probes can be found but they need less time to compute. Under "More relaxed design", more probes can be found but they require additional thermodynamic comparisons. These probe sets were computed either on a 2-CPU Opteron 2.0 GHz workstation or on a 4-CPU Opteron 2.2 GHz server with slightly higher memory overhead; thus, the computation time spent are comparable between the two machines (e.g., 10 minutes on the 2-CPU workstation is roughly equal to 5 minutes on the 4-CPU server). The data from this table are also drawn in Figure 6.

Another tradeoff is between the time PICKY spent on its computation and the number of probes it designed. This is illustrated in Figure 6 by using the data from Table 2. The CPU time and probe number difference between the two probe sets reported for each species are compared. For example, the smaller of the *E. coli *probe sets contains 4651 probes and was computed in 2 × 9 = 18 CPU-minutes. Its larger probe set has (4852-4651)/4651 = 4.3% more probes but takes (4 × 7-18)/28 = 36% more time to compute; thus, the 4% more probes are *expensive *because they require 36% more time to compute. When its design constraints are relaxed, PICKY has to consider more probe candidates, which require more thermodynamic comparisons and computation time. The two lines in Figure 6 indicate that generally more effort is needed to obtain new probes, i.e., the percentage time spent (red line) is often more than the extra probes gained (blue line). Notable exceptions of this trend are for maize, mouse and human. For these three species, more probes can be gained with relatively less computation time increase. It may be an anomaly with maize because its genome had not been fully sequenced at the time of this test, thus its gene set was limited to expressed sequences. With the mouse and human gene sets, however, this phenomenon suggests that they are more complex than the other genomes because their genes are similar at sequence level but still thermodynamically distinguishable. For example, increasing the nontarget match length from 15 to 17 and reducing the melting temperature difference from 20°C to 10°C doubled the time to evaluate the human gene set but almost tripled the number of probes that can be found by PICKY. From an information-theoretical point of view, this suggests that the mouse and human genomes have a higher information content -- the subtle differences among their genes can only be revealed by thorough thermodynamic comparisons [30]. The complexity and information content of a genome is not to be taken as its size, e.g., the *Arabidopsis *genome is about the same size as the *C. elegans *genome, but requires more time to process.

## Discussion

PICKY employs a comprehensive thermodynamics analysis to determine the similarity among gene sequences in order to design good microarray probes. This analysis helps PICKY find gene-specific probes [13,14]. In addition, the equations PICKY uses to determine thermodynamic characteristics are deterministic. The deterministic design approach in PICKY means that the commonly used blocking agents for nonspecific bindings such as human COT-1 DNA, yeast tRNA or salmon sperm DNA are not necessary when using PICKY designed microarrays. Unless their DNA sequences are included in the nontarget gene set given to PICKY during a microarray design, the blocking agents may actually degrade microarray data quality. Although many existing microarrays are not designed by PICKY, users can use PICKY to evaluate them and determine a subset of the probes to trust. In principle, this even works for microarray experiments that have already been completed; their results may be improved by filtering the data through the probe evaluation process using PICKY.

Although the shared probe design feature is developed for large genomes as a remedy when unique probes cannot be found for certain gene families, it is also possible to combine several gene sets and ask PICKY to design shared probes among different species. These shared probes can be used in comparative genomics [10], metagenomics (i.e., environmental sampling) [31] or pathogen identification [32]. With its default settings, PICKY minimizes probe sharing by first selecting unique probes, but PICKY can also be instructed to opt for probes that are shared by more target genes. The shared probe set from PICKY can then be minimized to detect several known species [33] or used in its entirety to detect as yet unknown species that are phylogenetically related to the species whose gene sets were used for the design [34]. For either application, the basic requirement is that a hybridization matrix *H *is given: the *H*_*ij *_entry is 1 if probe *j *can detect species *i *or 0 otherwise. In reality, microarray probes do not exhibit this binary behavior but vary their detection signal strength among different but related species. In this respect, the optimization of the melting temperature difference between targets and nontargets of all PICKY designed probes enhances their binary nature in detecting species (i.e., they can detect all target species with equal certainty but none of the nontargets). A recent quantitative evaluation of PICKY designed probes confirmed this characteristic [15].

## Conclusions

Shared probe design is a versatile feature that can increase detectable genes in large gene families and allow cross-genome microarrays to be developed. Usually, some genes sharing a probe also have their own unique probes; thus, by considering a combination of unique and shared probes we can still identify genes that lack unique probes to detect them. The nonlinear salt effect calculation expands the probe design sensitivity to another dimension, the salt concentration, and precisely matches the designed probes to specific microarray protocols and hybridization conditions. The microarray reanalysis function provides no-cost improvements to microarray data quality by utilizing improved genome annotations; this is not limited to microarrays designed by PICKY. An interesting future project will be to reanalyze some completed microarray projects by filtering their existing data through the PICKY reanalysis function to see if the statistical quality of the filtered data may be improved or some alternative conclusions may be drawn from the results.

## Authors' contributions

HC wrote the PICKY software and the manuscript. All authors read and approved the final manuscript.

## Availability and requirements

**Project name**: The PICKY oligo microarray design and analysis software

**Project home page**: http://www.complex.iastate.edu

**Operating system(s)**: Windows XP or later, Mac OS X 10.4 or later, and most Linux distributions running on ×86 compatible CPUs.

**Programming language**: C++

**Other requirements**: none

**License**: The PICKY project has never received public support and thus depends on commercial licensing fees to sustain its development and maintenance. Free academic licenses are provided to academic and nonprofit users after they execute the online license request and provide proof of their nonprofit status. Commercial users should contact PICKY http://picky@www.complex.iastate.edu to obtain commercial license information.

**Any restrictions to use by non-academics**: Commercial licenses required.
